# 5-EPIFAT trial protocol: a multi-center, randomized, placebo-controlled trial of the efficacy of pharmacotherapy for fatigue using methylphenidate, bupropion, ginseng, and amantadine in advanced cancer patients on active treatment

**DOI:** 10.1186/s13063-024-08078-w

**Published:** 2024-04-03

**Authors:** Mojtaba Miladinia, Mina Jahangiri, Sharon Jackson White, Hossein Karimpourian, Alessandro Inno, Sally Wai-Chi Chan, Reza Ganji, Mahmood Maniati, Kourosh Zarea, Marziyeh Ghalamkari, Ali Farahat, Cecilia Fagerström

**Affiliations:** 1 Nursing Care Research Center in Chronic Diseases, School of Nursing and Midwifery, Ahvaz Jundishapur University of Medical Sciences, Ahvaz, Iran; 2 Department of Biostatistics, Faculty of Medical Sciences, Tarbiat Modares University, Tehran, Iran; 3 School of Nursing, Clayton State University, Morrow, GA USA; 4 Department of Medical Oncology, School of Medicine, Ahvaz Jundishapur University of Medical Sciences, Ahvaz, Iran; 5 Medical Oncology Unit, IRCCS Ospedale Sacro Cuore Don Calabria, Negrar di Valpolicella (VR), Italy; 6 Tung Wah College, Kowloon, Hong Kong; 7 Department of Clinical Pharmacy, School of Pharmacy, Ahvaz Jundishapur University of Medical Sciences, Ahvaz, Iran; 8 School of Medicine, Ahvaz Jundishapur University of Medical Sciences, Ahvaz, Iran; 9 Department of Internal Medicine, School of Medicine, Iran University of Medical Sciences, Tehran, Iran; 10 Department of Hematology and Oncology, School of Medicine, Shahid Sadoughi University of Medical Sciences, Yazd, Iran; 11 Department of Health and Caring Sciences, Faculty of Health and Life Sciences, Linnaeus University, Kalmar/Växjö, Kalmar, Sweden; 12 School of Nursing and Midwifery, Deakin University, Burwood, VIC Australia

**Keywords:** Fatigue, Pharmacological treatment, Protocol, Randomized controlled trial, Study design

## Abstract

**Background:**

Cancer-related fatigue (CRF) is still undertreated in most patients, as evidence for pharmacological treatments is limited and conflicting. Also, the efficacy of the pharmacological agents relative to each other is still unclear. Therefore, medications that may potentially contribute to improving CRF will be investigated in this head-to-head trial. Our main objective is to compare the efficacy of methylphenidate vs. bupropion vs. ginseng vs. amantadine vs. placebo in patients with advanced cancer.

**Methods:**

The 5-EPIFAT study is a 5-arm, randomized, multi-blind, placebo-controlled, multicenter trial that will use a parallel-group design with an equal allocation ratio comparing the efficacy and safety of four medications (Methylphenidate vs. Bupropion vs. Ginseng vs. Amantadine) versus placebo for management of CRF. We will recruit 255 adult patients with advanced cancer who experience fatigue intensity ≥ 4 based on a 0–10 scale. The study period includes a 4-week intervention and a 4-week follow-up with repeated measurements over time. The primary outcome is the cancer-related fatigue level over time, which will be measured by the functional assessment of chronic illness therapy-fatigue (FACIT-F) scale. To evaluate safety, the secondary outcome is the symptomatic adverse events, which will be assessed using the Patient-Reported Outcomes version of the Common Terminology Criteria for Adverse Events in cancer clinical trials (PRO-CTCAE). Also, a subgroup analysis based on a decision tree-based machine learning algorithm will be employed for the clinical prediction of different agents in homogeneous subgroups.

**Discussion:**

The findings of the 5-EPIFAT trial could be helpful to guide clinical decision-making, personalization treatment approach, design of future trials, as well as the development of CRF management guidelines.

**Trial registration:**

IRCT.ir IRCT20150302021307N6. Registered on 13 May 2023.

**Supplementary Information:**

The online version contains supplementary material available at 10.1186/s13063-024-08078-w.

## Introduction

According to the clinical practice guideline of the National Comprehensive Cancer Network (NCCN), cancer-related fatigue (CRF) is defined as “a distressing, persistent, subjective sense of physical, emotional, and/or cognitive tiredness or exhaustion related to cancer or cancer treatment that is not proportional to recent activity and interferes with usual functioning” [1]. CRF affects 60 to 90% of cancer patients [2–4] and is the most common and debilitating symptom reported in people with advanced cancer [5]. It has been identified as a high-priority research area in the oncology setting (among the top 5) by the National Cancer Institute [6].

Although CRF is a complex, multidimensional and multifactorial problem that significantly affects patients’ quality of life and survival [7–11], it is still undertreated in most patients, mainly due to lack of any effective treatment [12]. Hence, CRF management is an important priority for patients and a serious challenge for clinicians in palliative cancer care [11, 13]. Although there are clinical guidelines for the management of CRF, it is still unclear which treatment method is most effective [14]. In general, treatment options for improving CRF are limited [15]. It has been shown that some non-pharmacological approaches (such as physical activity, massage therapy, etc.) are effective in controlling CRF. However, many patients do not have the desire or the ability to be committed to such treatments on a regular basis, and this is particularly true with patients with advanced cancer [16–18]. Therefore, pharmacological interventions can be considered a helpful approach, but evidence in this regard is limited and recommendations are often contradictory [19–21]. In fact, pharmacological strategies for the treatment of CRF have not yet been established, nor is there any consensus on pharmacological management [9, 19]. As a result, more research on pharmacological treatments of CRF is needed, especially in patients with advanced cancer [20]. Past trials have focused only on one pharmacological agent, and head-to-head clinical trials to compare the efficacy of different medications in CRF treatment are rare [15, 20, 22]. A Cochrane systematic review on pharmacological therapies for fatigue associated with palliative care strongly recommends that future trials should compare one anti-fatigue drug against another, along with a placebo [15].

Methylphenidate, bupropion, and ginseng have shown positive effects on improving CRF in some past trials [23–31]. Although these medications have been tested for years, their efficacy is still unclear, and there is no consensus among clinicians regarding their effectiveness in CRF treatment [15, 19, 20, 31]. Also, the above-cited Cochrane review study reports that amantadine appears to be promising in improving fatigue associated with multiple sclerosis, but whether this drug can also relieve fatigue in cancer patients has not been shown and should be investigated [15]. Therefore, the medications that will be tested in this trial are those that may potentially be effective in improving CRF.

To the best of our knowledge, no prior trial of pharmacological therapies for CRF has been conducted to compare the efficacy of different medications. Hence, we designed a head-to-head trial using subgroup analysis based on a decision tree-based machine learning algorithm for clinical prediction. The findings of the 5-EPIFAT trial could be helpful to guide clinical decision-making, personalize treatment approach, design future trials, and develop CRF management guidelines. The objectives of this study include [1] comparison of the efficacy of different pharmacological agents with placebo in managing CRF; [2] comparison of the efficacy of pharmacological agents with each other in managing CRF; [3] comparison of the efficacy of pharmacological agents in homogeneous subgroups of patients with CRF; and [4] comparison of the safety of treatment with pharmaceutical agents versus placebo and each other.

## Method

This 5-EPIFAT trial protocol is according to the Standard Protocol Items: Recommendations for Interventional Trial (SPIRIT) 2013 statement. All items from the WHO trial registry data set are found within the protocol accessible at https://irct.behdasht.gov.ir/trial/69613.

### Study design

This 5-EPIFAT study is a 5-arm, longitudinal, randomized, multi-blind, placebo-controlled, multicenter, superiority trial that will use a parallel-group design with an equal allocation ratio comparing the efficacy of four palliative medications (methylphenidate vs. bupropion vs. ginseng vs. amantadine) versus placebo in management of CRF among adult advanced cancer patients on active treatment. The study period will be 8 weeks (a 4-week intervention and a 4-week post-intervention follow-up) with repeated measurements over time.

### Settings

This trial will be conducted in 5 academic sites (3 hospitals and 2 outpatient oncology clinics) in 3 provinces of Iran (Khuzestan located in the southwest of Iran, Tehran located in the north-central Iran, and Yazd located in the center of Iran). More details of the study sites are available at https://irct.behdasht.gov.ir/trial/69613.

### Participants

A convenience sampling method will be used in this trial. Adult patients aged 18 years or older with advanced cancer (which is unlikely to be cured with treatment) who report moderate to severe CRF levels and are of different ethnicities and socio-economic status suffering from different cancer types will be included in the study. Eligibility criteria were set to maximize the generalizability of findings and prioritize participant safety. The eligibility criteria and withdrawal criteria are listed in Table 1.

**Table 1:** Inclusion, exclusion, and withdrawal criteria

**Inclusion criteria**	1) Age over 18 years old diagnosed with advanced cancer undergoing active anticancer treatment 2) Patients diagnosed with any type of cancer except CNS tumor, hormone-sensitive cancers, or pheochromocytoma 3) CRF diagnosis based on the International Classification of Diseases 10th Edition (ICD-10) 4) Reporting of moderate to severe fatigue in the last week (score ≥ 4 on a scale of 0 to 10) 5) Hemoglobin level higher than 9 g/dL in 2 weeks before enrollment 6) Ability to swallow and absorb medications 7) In case of the possibility of getting pregnant during the treatment and up to 6 weeks after, willingness to use effective contraceptive methods 8) Ability to read and write
**Exclusion criteria**	1) Presence of a known fatigue disorder not related to cancer 2) Presence of cognitive disorders, mental disorders (severe anxiety, major depression, schizophrenia, bipolar syndrome), neurological or brain disorders (dementia, delirium, Tourette syndrome, motor tics, epilepsy, history of stroke, aneurysm), diabetes, untreated severe anemia or anemia that requires blood transfusion, severe and uncontrolled pain and insomnia, serious cardiac disorders, uncontrolled arrhythmia or hypertension, history of long QT syndrome, glaucoma, intestinal obstruction, uncontrolled hypothyroidism, respiratory disorders that limit participation, autoimmune diseases, bleeding disorders 3) Abnormal function of the liver (high ALT or AST) and kidney (abnormal Cr or GFR less than 50) 4) History of major surgery in 1 month before enrollment 5) Taking erythropoietin, psychostimulants, antidepressants, food supplements, or other drugs to control fatigue currently or in the 4 weeks before participating in the study 6) Simultaneous use of drugs (including warfarin, anticonvulsants, tricyclic antidepressants, antipsychotics, monoamine oxidase inhibitors, clonidine, theophylline, caffeine and pseudoephedrine) 7) Major dose change (more than 25%) of opioids in 48 h before enrollment 8) Hypersensitivity to sympathomimetic amines 9) Planned surgery within 2 months of screening 10) History of sensitivity to or intolerance of the medications under study 11) Pregnant or lactating women 12) History of drug or alcohol abuse in the past year 13) Involvement in other clinical trials
**Withdrawal (discontinuation) criteria**	1) Voluntary withdrawal of the trial by the patient for any reason 2) Significant deterioration in the patient’s clinical condition 3) Occurrence of severe/serious adverse event(s) 4) The need to take erythropoietin during the study period 5) Patient or doctor recognition that stopping the medications is in the patient’s best interest

### Intervention

**Fig. 1:**
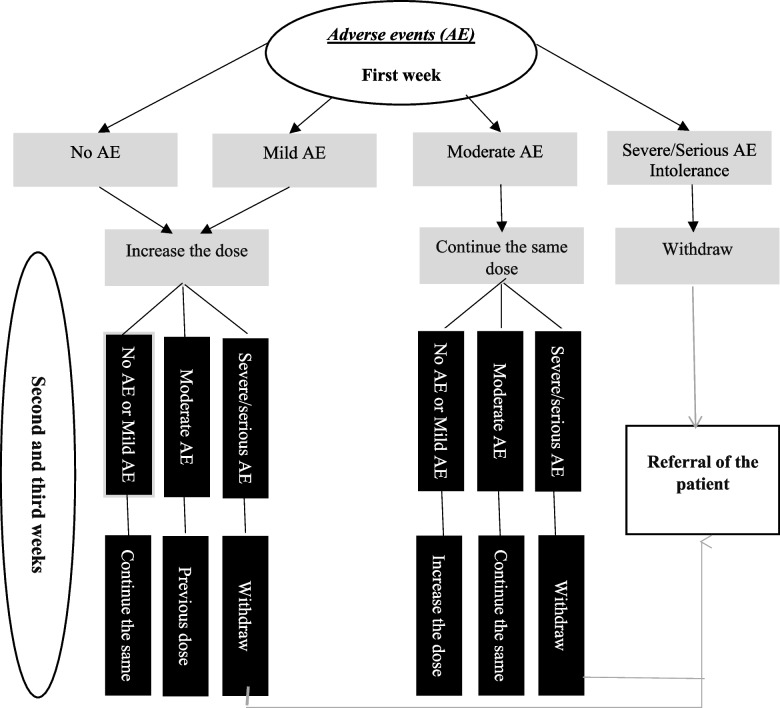
Algorithm guiding nurses who contact patients regarding dosage adjustment

In this study, four groups will receive medicine, and one group will be given placebo for 4 weeks. All medications will be prepared in opaque and identical (in terms of size, shape, and color) capsules by the study pharmacist, and then they will be packed in sufficient quantities in opaque and identical cans. In all groups, the participants will start taking one capsule in the morning within the first week. After that, in case the participants tolerate the medication well and do not report significant adverse events (AEs) attributed to the medication, they will take two capsules daily (morning and evening) in the second and third weeks. Afterwards, in the fourth week, one capsule will be given to the participants in the morning in order to reduce the possibility of withdrawal symptoms. Figure 1 shows the guiding algorithm of nurses who will contact participants regarding medication dosage adjustment. All AEs will be handled appropriately. In case of severe or serious AEs, the medication will be discontinued, and the participant will be withdrawn from further treatment. The criteria for discontinuing allocated intervention are listed in Table 1.

The study arms include the following: Arm 1, study participants will receive oral methylphenidate for 4 weeks. Each capsule contains 10 mg of methylphenidate. Methylphenidate will be started in the first week with a dose of 10 mg daily (in the morning). If the patient tolerates it well, the dose of 10 mg twice a day (morning and evening) will be continued in the second and third weeks. Then, in the fourth week, the dose is again reduced to 10 mg daily (in the morning) to reduce the possibility of withdrawal symptoms; Arm 2, study participants will receive oral bupropion sustained-release for 4 weeks. Each capsule contains 150 mg of bupropion. Bupropion will be started in the first week with a dose of 150 mg daily (in the morning). If the patient tolerates it well, the dose of 150 mg twice a day (morning and evening) will be continued in the second and third weeks. Then, in the fourth week, the dose is again reduced to 150 mg daily (in the morning) to reduce the possibility of withdrawal symptoms; Arm 3, study participants will receive oral amantadine for 4 weeks. Each capsule contains 100 mg of amantadine. Amantadine will be started in the first week with a dose of 100 mg daily (in the morning). If the patient tolerates it well, the dose of 100 mg twice a day (morning and evening) will be continued in the second and third weeks. Then, in the fourth week, the dose is again reduced to 100 mg daily (in the morning) to reduce the possibility of withdrawal symptoms; Arm 4, study participants will receive oral Panax ginseng for 4 weeks. Each capsule contains 500 mg of ginseng. Ginseng will be started in the first week with a dose of 500 mg daily (in the morning). If the patient tolerates it well, the dose of 500 mg twice a day (morning and evening) will be continued in the second and third weeks. Then, in the fourth week, the dose returns to 500 mg daily (in the morning); Arm 5, study participants will receive an oral placebo. The placebo will be started in the first week with one capsule daily (in the morning). After that, two capsules daily (morning and evening) will be given to patients in the second and third weeks. Then, in the fourth week, the patients will again receive one capsule (in the morning). All patients will receive usual care and treatment regimens during the study.

#### Pharmacological agents

Although methylphenidate, bupropion, and ginseng have been investigated in various studies for the management of CRF, the findings are contradictory, and their clinical effectiveness is still in doubt and requires further research [12, 15, 19, 20]. Some studies have also shown that amantadine has promising effectiveness in improving fatigue associated with chronic diseases, but this needs to be investigated in cancer patients as well [15]. Therefore, the medications that will be evaluated in this 5-EPIFAT trial are those that we assume will be effective in improving CRF. Also, due to the uncertain effectiveness of the medications in this trial, we also included a placebo group. Comparing the effectiveness of these agents against each other requires showing their superiority over the placebo in the first place, and then over each other in the second place. In Table 2, a brief review of the medications selected for the 5-EPIFAT study is given.

**Table 2:** Pharmacological agents selected in the 5-EPIFAT trial

Pharmacological agents	Description
**Methylphenidate**	Methylphenidate is a psychostimulant which has been frequently studied and is widely prescribed for the medical management of patients with attention deficit hyperactivity disorder [3, 32]. Methylphenidate is considered as one of the promising pharmacological options in improving CRF, which is well tolerated by patients, although the results about its efficacy are mixed [29]. Both NCCN and ASCO recommend psychostimulant drugs (such as methylphenidate) as pharmacological options in active treatment patients, although they note that clinical evidence is limited [33]
**Bupropion**	Bupropion is a non-serotonergic antidepressant that may be useful in the treatment of CRF. Bupropion is widely available as a generic drug with an excellent safety profile, and there have been frequent requests to investigate bupropion as a pharmacological agent for the treatment of CRF [13]. Bupropion is an antidepressant with a dual effect on the neurotransmitter systems of norepinephrine and dopamine, so psychopharmacologically it shares a wide range of actions with psychostimulants [25]
**Ginseng**	Thanks to its various pharmacological effects, ginseng is one of the most valuable herbs in herbal medicine. Ginseng is widely used in the United States and other countries, based on the belief that it improves energy levels, and relieves stress and mental and physical fatigue [34]. Panax Ginseng extract has a direct effect on the central nervous system, and the ability to modulate inflammatory cytokines. Despite its frequent use, evidence for its effect on the improvement of CRF is limited [35]. Ginseng is a good treatment option, especially in patients who want to use herbal medicines
**Amantadine**	Amantadine is a drug approved by the Food and Drug Administration for the prevention of influenza and the symptomatic treatment of Parkinson’s disease, and it is the most widely studied drug for fatigue associated with multiple sclerosis [36]. It is unclear which pharmacological effect may be responsible for the possible anti-fatigue properties of amantadine [37]. According to a Cochrane systematic review study, amantadine appears to be promising in improving fatigue in other chronic conditions, but whether or not it relieves fatigue in cancer patients has not been shown, and should be investigated [15]

*Abbreviations*: *CRF*, cancer-related fatigue; *NCCN*, National Comprehensive Cancer Network; *ASCO*, American Society of Clinical Oncology

### Outcomes and data collection

The tools will be given to patients in the form of a diary. Demographic and clinical data will be collected using a researcher-developed checklist at baseline (before random allocation). To evaluate the efficacy, the main outcome is the cancer-related fatigue level over time, which will be measured using the Functional Assessment of Chronic Illness Therapy-Fatigue (FACIT-F) self-reported scale version 4 [38]. CRF level will be measured at baseline, weekly during the 4-week intervention period, and at weeks 6 and 8 as a follow-up in order to evaluate the potential durability of the effect of each medication on improving fatigue [The intervention termination is the 4th week, and follow-up termination is the 8th week]. We will compare the trend of changes in mean fatigue level over time (from baseline to the eighth week) between the trial arms. FACIT-F is a multidimensional and validated patient-reported outcome that includes 13 questions with a recall period of 7 days. Questions are scored based on a Likert scale from 0 to 4 (from “not at all” to “very much”). To score the FACIT-F, all questions are summed up to create a single fatigue score ranging from 0 to 52, and negative items are reversely scored. Higher scores represent better results (i.e., less fatigue). The FACIT-F has been widely applied in various cancer populations, and results demonstrate satisfactorily psychometric properties [38, 39]. We obtained the license for using FACIT in this study. Considering that the intensity of fatigue varies at different times of the day, we will ask all participants to complete the questionnaire at a certain time of the day (i.e., between 2 and 6 pm).

To evaluate safety, the secondary outcome is the symptomatic adverse events, which will be assessed using the Patient-Reported Outcomes version of the Common Terminology Criteria for Adverse Events in cancer clinical trials (PRO-CTCAE) [40]. PRO-CTCAE is a validated tool that measures the frequency, severity, or interference of symptoms experienced by patients participating in cancer clinical trials. This tool is designed to assess symptomatic toxicities experienced by patients. The frequency, intensity, interference, degree, and presence of 78 symptomatic side effects are evaluated in this tool. The recall period of this tool is 1 week, which also matches well with its daily report, so it will be completed weekly by the patients [40, 41]. PRO-CTCAE will be completed at baseline, and weekly during the 4-week intervention period. We will compare the frequency of adverse events between the trial arms from week 1 to week 4. Safety analysis will be performed for all patients who receive at least one dose of the medication. The pharmacological agents studied in this trial have known safety profiles and are already approved and used for other conditions. Therefore, we do not need laboratory indicators and clinical examinations to diagnose side effects, and we will simply collect patient-reported adverse events.

### Trial handling

**Fig. 2:**
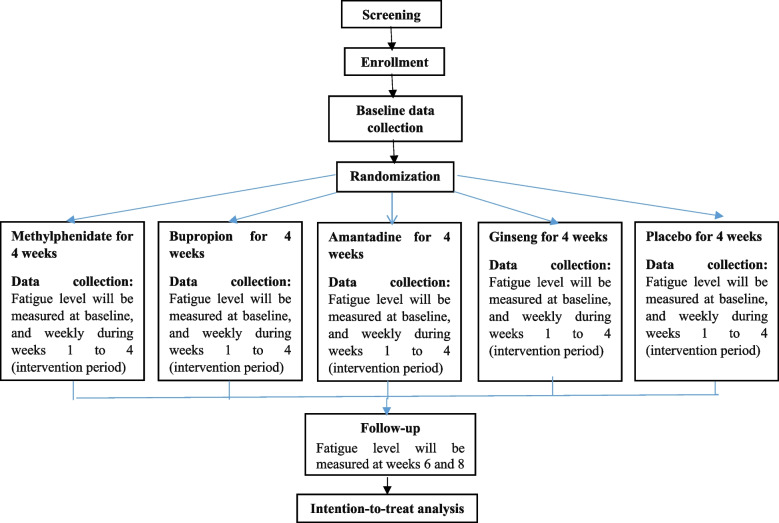
The step by step process of 5-EPIFAT trial

In this study, blinded nurses will make phone calls to all patients to remind them to complete the diary and adjust the drug dose in weeks 1 to 4 (with a weekly schedule) and weeks 6 and 8 (or more if necessary). Also, during the second and fourth weeks, the patients will be visited in-person only for the purpose of checking how they complete the diary and take the medications and ensuring their safety (no data collection will be done during the in-person visits). Nurses are required to determine the reasons for participants who discontinue or deviate from intervention protocols and record them in the checklist. At the end of the eighth week, the data diaries will be collected and imported into the statistical software. Also, the data entry process will be periodically double-checked by the statistician to prevent registration errors. The trial implementation process is shown step by step in Fig. 2.

### Recruitment

Eligible patients will be continuously recruited until the target sample size is reached. Oncologists and oncology nurses will screen patients at 5 sites, and refer the patients who meet the initial criteria. Then, the patients will be subjected to detailed evaluation (including demographic and clinical characteristics, history, and medical record) by trained study staff for final confirmation of eligibility, obtain written informed consent, and collect baseline data.

### Assignment and blinding

A permuted block randomization allocation algorithm with an equal allocation ratio (1:1:1:1:1) will be generated by the statistician before enrollment using the package blockrand [42]. The statistician will then send the randomization list to the study pharmacist and randomization provider. The pharmacist will not have any role whatsoever in data collection or analysis. Neither will the randomization provider play a role in the rest of the trial. Each participant giving written consent to participate in the study will be identified with a unique two-part hash code. This code is a combination of the unique number of each center and the patient’s number. In each center, the first recruited patient will be assigned number 1, and consecutive numbers will be assigned to the next participants. At each site, a blinded research assistant will send a randomization request to the randomization provider, who will immediately receive the order via email, assign the participants to one of the 5 study groups (identified by a code), and provide them with the medication package (these assistants will not be involved in the rest of the trial). Other personnel of the research team will be blind until the end of data analysis. Also, for the purpose of blinding, the medications will be placed by the study pharmacist in completely identical opaque capsules in similar opaque boxes differently coded with preprinted medication code labels (a specific 3-letter code will be considered for each of the five medications used in the study). The medication codes and randomization list will be kept by the study pharmacist until the trial is closed (only in case of a serious adverse event, the patient’s medication code will be broken). The exact details of blinding are listed in Table 3.

**Table 3:** Blinding/masking details

Individuals	Information withheld	Method of blinding	Considerations
**Participants**	Group assignment and study hypotheses	Similar appearance and packaging of medications	Participants will be unblinded through email after the trial is closed
**Principal investigators**	Group assignment	- Concealed allocation schedule - Similar medications and packaging	They will be blinded until the end of the data analysis
**Randomization provider**	Not blinded	-	She will not be involved in the rest of the trial
**Research assistants assigning participants**	Group assignment, purposes and hypotheses of the study, and participant characteristics	- Concealed allocation schedule	They will not be involved in the rest of the trial
**Pharmacist**	Not blinded	-	The pharmacist will not have a role in data analysis
**Statistician**	Group assignment, participant, and group identities	Codes are given to participants and groups	-
**Nurses who make contact with participants**	Group assignment, study hypotheses, and participant characteristics	- Concealed allocation schedule - Participants are given numerical identifiers	They will not have any role in data analysis or manuscript writing
**Data collectors**	Not applicable	-	The data are patient-reported outcomes
**Manuscript writers**	Not applicable	-	-
**Data and Safety Monitoring Committee (DSMC)**	Group assignment	- Concealed allocation schedule	They can request to break the code for any participant at any time if needed

### Participant timeline

Table 4 shows the details of the participant timeline (SPIRIT diagram).

**Table 4:** SPIRIT diagram (Standard Protocol Items: Recommendations for Interventional Trials**)**

Study period										
	**Enrolment**	**Allocation**	**Intervention** **(Post-allocation)**				**Follow-up** **(Post-allocation)**			
**Timepoint**	*** − t***_***1***_	**0**	***Week 1***	***Week 2***	***Week 3***	***Week 4***	***Week 5***	***Week 6***	***Week 7***	***Week 8*** ***(Close-out)***
**Enrolment**										
*- Eligibility screen*	X									
*- Informed consent*	X									
***- Random allocation***		X								
**Interventions:**										
*- Pharmaceutical groups*			X	X	X	X				
*- Placebo group*			X	X	X	X				
**Assessments:**										
*- Baseline data collection*	X									
*- Main outcome (fatigue level)*	X		X	X	X	X		X		X
*- Secondary outcome (PRO-CTCAE questionnaire for adverse events)*	X		X	X	X	X				
*- Nurse phone calls*			X	X	X	X		X		X
*- In-person visits*	X			X		X				

*Abbreviation: PRO-CTCAE*, Patient-Reported Outcomes version of the Common Terminology Criteria for Adverse Events

### Sample size

Using the package WebPower in R software, a sample size of 205 subjects was calculated based on comparing study groups assuming a repeated measures ANOVA with a 5% level of significance, 80% power, an effect size equal to 0.57 [31], the number of groups (5-arm), and the number of repeated measures (7 time points) [43]. Finally, assuming a drop-out rate of 20%, the sample size was set to be 255 subjects who were supposed to be randomly assigned in each arm (*n* = 51 each).

### Statistical methods

#### Analysis plan

Descriptive statistics of qualitative and quantitative variables will be shown as frequency (percentage) and mean ± standard deviation (SD), respectively. Analysis of variance (ANOVA) and Kruskal–Wallis test will be used to assess the difference between quantitative variables across study groups (including age and body mass index). Chi-square test and trend chi-square test will be used to test the difference of frequency distribution of qualitative variables across study groups (including sex, ethnicity, type of cancer, and type of treatment). The normality of data will be evaluated using the Shapiro–Wilk test. Two-tailed *p*-values less than 0·05 will be regarded as statistically significant, and data analysis will be done using free statistical software R. The interpretation of the results will be based on intention-to-treat (ITT) analysis.

##### Primary outcome (fatigue level)

If there is a significant interaction effect between the study groups and the time factor to assess the changes in the primary outcome variable over time (from the baseline to the eighth week), then the study groups will be compared within each time point using ANOVA based on the Bonferroni correction to adjust the type I error. Since the primary outcome variable does not have a normal distribution, the changes over time points will be assessed using nonparametric repeated measure analysis in the nparLD package [44]. In addition, if there were not the same number of measurements for each participant over time, we would use the linear mixed-effect model to compare the treatment groups using the lme4 package because this model can deal with any degree of imbalance in the longitudinal data [45]. The waterfall and spider plots will be used to represent each individual patient’s response and the individual changes in response over time relative to baseline using the waterfall and ggplot2 packages, respectively [46–48]. All time points will be used for analysis.

##### Secondary outcome (adverse events)

The assessment of safety will be based on the frequency of adverse events from week 1 to week 4 (number [percentage] of participants reporting each type of event in each subgroup of adverse events), and comparison between groups will be made using chi-square test.

#### Subgroup analysis

The linear mixed-effect model tree (LMEMT) using the glmertree package will be employed to predict the efficacy of medications in different subgroups. This method is a modern flexible tree algorithm for subgroups in longitudinal data [49]. Tree-based algorithms are supervised non-parametric algorithms and are one of the most popular machine learning tools for modeling and clinical prediction. These methods have some notable advantages over parametric models such as easy interpretability due to the graphical display of the results, which is one of the attractive properties of tree models, without the need to determine assumptions about the functional form of the data, deal with nonlinear relationships and high-order interactions, and extract homogeneous subgroups of observations. As a powerful method of machine learning, tree algorithms can identify homogeneous subgroups of patients who need different treatment strategies, and they can also guide the clinician in decision-making [50].

#### Missing data

Longitudinal data collected from the same subjects over time is frequently used in clinical trials. In these studies, the researchers often encounter major challenges including missing values. To alleviate this problem, we will use the Copy Mean method to impute monotone/dropout and non-monotone/intermittent missing data of longitudinal quantitative response variables [51, 52]. To understand the computational strategy related to this method, a time-dependent variable is recorded at *t* time points for each subject. In this case, a trajectory for cluster *i* is defined as the sequence *y*_*i*._ = (*y*_*i*1_, *y*_*i*2_, …, *y*_*it*_). Let *y*_*ik*_ show a missing value for cluster *i* at a specific time point *k*. *y*_*ik*_ is non-monotone missing if time points as *a* < *k* < *b* exist and *y*_*ia*_ and *y*_*ib*_ are not missing. *y*_*ik*_ is monotone missing if for all time points *h* > *k*, *y*_*ih*_ is missing. The copy-mean method is based on two steps for the imputation of missing observations. First, the missing values are imputed using the LOCF method to provide an initial approximation of these values (In the LOCF method, *y*_*ik*_ is estimated by the last observed value of the trajectory of interest). Then the mean trajectory of the population is used to refine the initial approximation in the previous step. Let,

(${\overline{y} }_{.1}$, …, ${\overline{y} }_{.t}$): the mean trajectory of a population.

*y*_*ik*_: the first missing value of *i*th trajectory.

*y*_*ik*_^LOCF^: the imputed value for *y*_*ik*_ using LOCF method for all time points *k* ≥ *d.*

(${\overline{y} }_{.1}$ ^LOCF^, …, ${\overline{y} }_{.t}$ ^LOCF^): the mean trajectory of a population with missing values using the LOCF method.

AV_*k*_: the average variation at *k*th time point and is equal to ${\overline{y} }_{.k}$ ‒ ${\overline{y} }_{.k}$ ^LOCF^

The missing value *y*_*ik*_ is obtained from the Copy Mean LOCF by adding AV_*k*_ to the imputed value for *y*_*ik*_ using the LOCF method (*y*_*ik*_^LOCF^ + AV_*k*_).

#### Interim analyses

No interim analyses of efficacy towards the primary endpoint will be performed because we do not expect any of the trial medications to have dramatic effects compared with other medications.

### Ethics, monitoring, and responsibilities

This study was registered in IRCT.ir on 13/05/2023, https://irct.behdasht.gov.ir/trial/69613. The 5-EPIFAT protocol is approved by the Institutional Review Board (IRB) of Ahvaz Jundishapur University of Medical Sciences (Ref. ID: IR.AJUMS.REC.1401.587). All sites received local approval from their IRBs. The trial will be evaluated by an independent Data and Safety Monitoring Committee (DSMC). DSMC members will be selected by the ethics committee and will independently monitor the entire trial process and data. The DSMC will conduct both on-site and remote monitoring. The DSMB and the IRB must approve any modifications to the protocol. Amendments are first notified to the funder, the IRB, and the DSMC. When approved, the PI will notify the centers, and a copy of the revised protocol will be sent to the PI to add to the Investigator Site File. Also, amendments will be updated in the IRCT clinical trial registry. To double-check the details, go over the timeline table, and finally approve the protocol, the principal investigators (PI), DSMC, and IRB representative attended the first open meeting. Regular DSMC meetings will be held at quarterly intervals during the trial period. The Site Principal Investigator (PI) is responsible for leading the clinical trial team, overseeing the conduct of clinical trial at a site in accordance with the approved protocol, and is responsible for adequately supervising the team members. Also, the PIs are responsible for monitoring and coordinating the process of gathering, entering, safeguarding data during the trial. The PIs must report any potential protocol deviations and the incidence of serious adverse events to the main site within 24 h. Trained study staff are responsible for running the trial according to the protocol, which are monitored regularly and weekly by the PI or their representative at each study site. In case of any severe adverse event, the DSMC will be notified within 48 h by a short statement and then a fully detailed report will be prepared. In addition, the patient is referred for side effects control free of charge. The electronic version of the data will be encrypted and stored with a double password, and only the main authors (MM, HK, MJ) will have access to the dataset. Finally, the 5-EPIFAT trial data will be presented in peer-reviewed journals and at national and international conferences. The protocol is accessible in IRCT.ir. Data and statistical codes will be available upon reasonable request from the corresponding author.

## Discussion

Fatigue is one of the most distressing and common symptoms experienced by cancer patients, and its management poses serious challenges for medical oncologists, nurses, and palliative care specialists. Treatment options for cancer-related fatigue are still emerging [53]. Overall, the evidence for the use of pharmacological agents for CRF is still not very solid [20]. Because the exact pathophysiological mechanism of CRF is not known, it is difficult to find effective pharmacological treatments for managing CRF [53]. Therefore, more clinical studies are needed to find palliative pharmacotherapy options for the treatment of CRF, especially studies that compare the effectiveness of different drugs. More particularly, further clinical evidence is still needed concerning the efficacy of palliative pharmacotherapy for treating CRF, particularly studies comparing the efficacy of different medications [27]. The 5-EPIFAT trial is the first multi-arm, head-to-head randomized trial for CRF treatment, comparing the efficacy of methylphenidate vs. bupropion vs. ginseng vs. amantadine vs. placebo in patients with advanced cancer on active treatment phase.

The 5-EPIFAT study is a multi-arm trial. Evaluating more than one intervention simultaneously increases the chance of finding an effective intervention. The use of a multi-arm design, as opposed to 2-arm trials, provides the possibility of comparing the effectiveness of different interventions with each other [33, 54]. The 5-EPIFAT study has a repeated measures design by virtue of which the efficacy of medications could be observed, which increases statistical power [55].

Conducting a trial with a cross-over design will be time-consuming due to the multi-arm nature of the study, and due to the possibility of some medications having no effectiveness compared to the placebo, the attrition rate is expected to be high in some groups, and there will be bias. On the other hand, although the agents used to have a short half-life and a cross-over design requires a short wash-out period, it is possible that the medications effective in improving fatigue have a long-lasting effect and the patient experiences less fatigue long after stopping the drug. Therefore, there is concern about carryover effects even if the wash-out period is taken into consideration. However, a parallel-group design does not have the mentioned problems and is more suitable.

All the medications used in this trial have a rapid onset of possible effects, so they are comparable with each other, and a 4-week period of use is sufficient to see their effects. In order to have a more realistic world, we will set relatively broad inclusion criteria, and the exclusion criteria will be chosen with the aim of patient safety. Different types of cancer will be taken into account because CRF is a syndrome that results from increased inflammatory cytokines and tumor byproducts regardless of tumor type. This pathophysiology of CRF is more related to the interaction between the cancer and the host than to any specific histology. Also, the frequency and severity of CRF in patients with different types of tumors are to a large extent the same [35].

In addition, the 5-EPIFAT trial will detect treatment-subgroup interactions with subgroup analysis using a decision-tree-based machine learning method [49] to guide clinical decision-making for CRF management in order to achieve the best effectiveness.

We expect that this study will lead to future evidence-based treatment of CRF in patients with advanced cancer, and to the design of clinical trials and guidelines. Even if the findings are negative, the results of this study will still be critical for further efforts to elucidate the pathophysiology of new therapeutic targets for CRF.

### Trial status

Participant recruitment commenced on September 2023 (protocol version 2, dated August 2023) and is ongoing. Up to now, 45 participants have been recruited. Recruitment is expected to be completed in September 2024.

### Statement of patient and public involvement

The public and patients were not consulted about how research protocols are written. At the conclusion of the study, social media will be used to share the findings with the patients.

### Declaration of authorship

The standard criteria to determine authorship for publishing our trial results will have significant contributions to the work’s conception or design, or to the gathering, processing, or interpretation of data; composing the work or critically editing it to remove any significant intellectual content; and the final approval of the version to be published, along with a commitment to take responsibility for every aspect of the work and make sure that any concerns about the integrity or accuracy of any part of it are duly looked into and addressed. Our team is dedicated to transparently discussing and reaching consensus on the distinct contributions of every possible author at the beginning of the research phase. We will guarantee that all individuals meeting the specified criteria are acknowledged as authors. Furthermore, individuals who assist in the project but do not qualify for authorship will be appropriately recognized in the acknowledgments section of the publication.

### Supplementary Information

**Supplementary Material 1.**

## Data Availability

The final dataset and the code for statistical analysis will be available upon reasonable request.

## Abbreviations

CRF: Cancer-related fatigue
NCCN: National Comprehensive Cancer Network
AE: Adverse event
ASCO: American Society of Clinical Oncology
FACIT-F: Functional assessment of chronic illness therapy-fatigue
PRO-CTCAE: Patient-Reported Outcomes version of the Common Terminology Criteria for Adverse Events
SPIRIT: Standard Protocol Items: Recommendations for Interventional Trials
DSMC: Data and Safety Monitoring Committee
PI: Principal investigator
